# Effectiveness of mepolizumab therapy on symptoms, asthma exacerbations, steroid dependence, and small airways in patients with severe eosinophilic asthma

**DOI:** 10.3906/sag-2009-41

**Published:** 2021-08-30

**Authors:** İnsu YILMAZ, Sakine NAZİK BAHÇECİOĞLU, Murat TÜRK, Nuri TUTAR, Gülden PAÇACI ÇETİN, Bahar ARSLAN

**Affiliations:** 1 Division of Immunology and Allergy, Department of Chest Diseases, Faculty of Medicine, Erciyes University, Kayseri Turkey; 2 Department of Immunology and Allergy, Atatürk Chest Disease and Thoracic Surgery Training and Research Hospital, Ankara Turkey; 3 Department of Immunology and Allergy, Kayseri City Training and Research Hospital, Kayseri Turkey; 4 Department of Chest Diseases, Faculty of Medicine, Erciyes University, Kayseri Turkey

**Keywords:** Mepolizumab, severe eosinophilic asthma, small airways, pulmonary function, asthma control test, oral corticosteroids

## Abstract

**Background/aim:**

The efficacy of mepolizumab has been largely demonstrated in clinical trials in patients with severe eosinophilic asthma (SEA). However, reports on experience with mepolizumab in a real-life cohort are limited. Moreover, data about the effectiveness of mepolizumab on small airways is scarce. This study evaluated the effectiveness of mepolizumab therapy on symptoms, asthma exacerbations, blood eosinophils, steroid dependence, and small airways in a real-life cohort of patients with SEA.

**Materials and methods:**

We retrospectively analyzed patients with SEA who were receiving fixed-dose mepolizumab. The effects of mepolizumab on clinical, laboratory, functional parameters were evaluated at 12th, 24th, and 52nd weeks. Small airways were assessed with the FEF 25-75.

**Results:**

A total of 41 patients were enrolled in the study. Mepolizumab significantly reduced asthma exacerbation rates, reduced mOCS dose, and improved asthma control test (ACT) scores at 12th, 24th, and 52nd weeks. However, we found no significant changes in FEV_1_ and FEF25-75 values at baseline, 12th, 24th, and 52nd weeks (78.9 ± 23.3%, 82.9 ± 23.4%, 81.9 ± 23.9%, and 78.9 ± 23.5% for FEV1; 45.1 ± 23.1%, 48.8 ± 23.5%, 48.7 ± 23.1%, and 41.0 ± 20.1% for FEF25-75, respectively)

**Conclusion:**

In this study, mepolizumab significantly improved all outcomes (symptom scores, asthma exacerbations, OCS sparing, and blood eosinophils) except functional parameters. Still, despite the dose reduction in mOCS dosage, no significant deterioration was observed in FEV_1_ and FEF25-75 values.

## 1. Introduction

Eosinophilic asthma generally refers to the clinical inflammatory phenotype of asthma wherein a significant number of sputum, airway, and/or blood eosinophils are present [1]. Eosinophils are key effector cells in bronchial inflammation and represent one of the main targets for biological agents. Interleukin-5 (IL-5) is the pivotal cytokine responsible for the maturation, activation, proliferation and survival of eosinophils [2,3]. Therefore, IL-5 represents a suitable specific target for biological treatments of severe eosinophilic asthma (SEA). Mepolizumab is a humanized IgG1/k monoclonal antibody which targets human IL-5 and thus prevents its interaction with the α chain of the IL-5 receptor [4,5]. 

Previous effectiveness studies of mepolizumab have clearly demonstrated that mepolizumab caused a meaningful lowering effect on blood eosinophils, was able to reduce asthma exacerbation rates, had a significant glucocorticoid-sparing effect and improved symptom control in asthma [6–9]. On the contrary, data regarding post-marketing studies that have evaluated the effects of mepolizumab in real-life settings are limited. Furthermore, the data about the effectiveness of mepolizumab therapy on small airways is quite limited in patients with SEA. Therefore, we evaluated effectiveness of mepolizumab on symptoms, asthma exacerbations, blood eosinophils, steroid dependence, and small airways in the real-life settings. 

## 2. Materials and methods 

This retrospective study included 41 severe asthmatics who had been treated with mepolizumab between 2018 and 2020. All patients were treated with high-dose inhaled glucocorticoids (ICS) (extrafine hydrofluoroalkane-beclomethasone dipropionate), and a long-acting β_2_-agonist, along with a second controller montelukast at least 6 months and most of patients were receiving mOCS therapy before the mepolizumab treatment. Indications to be treated with mepolizumab were approved on the basis of the Turkey Social Security Institution Health Application Communique, according to which, mepolizumab can be administered to patients with SEA having: a) blood eosinophil count ≥300 cells/µL (≥150 cells/µL: If the patient is under long-term regular OCS therapy); b) controlled or uncontrolled asthma treated with regular systemic steroids for at least 6 months and/or uncontrolled asthma (relatively two attacks per year requiring systemic corticosteroids for at least 3 days) despite use of a high combination dosage of ICS (> 800 mcg/day budesonide or equivalent) and inhaler long-acting β_2_ agonist for at least one year [10].^1^Turkey Social Security Institution Health Application Communique. [Online]. Websıte: https://www.mevzuat.gov.tr/mevzuat?MevzuatNo=17229&MevzuatTur=9&MevzuatTertip=5 [September, 2020].

**Figure 1:**
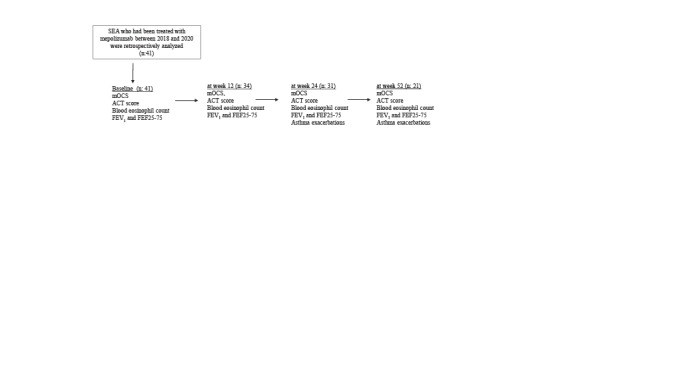
Effects of mepolizumab on clinical, laboratory, and functional parameters were evaluated at 12th, 24th, 52nd weeks. Small airways were assessed with the FEF25-75.

Throughout the study period, parameters including mOCS (presented as methyl-prednisone equivalent in milligrams), asthma control test (ACT) score, blood eosinophil count, forced expiratory volume in 1 s (FEV_1_) and FEF25-75 were measured at baseline, at week 12, at week 24, and at week 52 after the first injection of mepolizumab. In addition, the numbers of asthma exacerbations were also recorded at baseline, week 24, and week 52 (Figure 1). 

All patients were under follow-up at our asthma outpatient clinic provided written informed consent. Ethical approval was obtained from the Erciyes University Ethics Committee.

### 2.1. Definitions

#### 2.1.1. Treatment response to mepolizumab

Mepolizumab was continued if there was a clinical response at week 12. Responder: ACT variations were higher than the accepted minimal clinically important differences of three points in real life [10]**. **Eligibility of patients to continue treatment was based on an increased in ACT score of ≥3 from the baseline or clinically significant reduced dose of mOCS without deterioration in ACT or reduction of exacerbation rate by at least 50%. 

#### 2.1.2. Asthma exacerbations

An exacerbation was defined as worsening of asthma symptoms, requiring OCS at least three days a week or an increase in the mOCS dose. 

#### 2.1.3. Chronic rhinosinusitis with nasal polyposis (CRSwNP)

CRSwNP is characterized by the occurrence for more than 12 weeks of symptoms as nasal discharge, stuffiness, facial pressure or pain, dysfunction or loss of the sense of smell, and cough from post-nasal drip. The polypoid inflammation filling the nasal airway in the paranasal sinus computerized tomography (PNCT) [11,12]. 

### 2.2. Glucocorticoid reduction phase scheme

The dose of methylprednisone was reduced every four weeks according to a predefined schedule (Table 1), if the patient did not have an exacerbation with a decrease in ACT score. In patients who were receiving a daily dose of 8 mg or more of methylprednisone at baseline, the dose of the drug was not reduced to 0 without consulting to endocrinology due to concern regarding withdrawal effects.

**Table 1:** Glucocorticoid reduction phase scheme.

Methylprednisolone dose (mg/day)						
20.0	16.0	12.0	10.0	8.0	6.0	4.0
16.0	12.0	10.0	8.0	6.0	4.0	2.0
12.0	10.0	8.0	6.0	4.0	2.0	2.0*
10.0	8.0	6.0	4.0	2.0	2.0*	0.0
8.0	6.0	4.0	2.0	2.0*	0.0	0.0
6.0	4.0	2.0	2.0*	0.0	0.0	0.0
4.0	2.0	2.0*	0.0	0.0	0.0	0.0
2.0	2.0*	0.0	0.0	0.0	0.0	0.0
2.0*	0.0	0.0	0.0	0.0	0.0	0.0

*Taken as 2.0 mg administered every other day.

### 2.3. Pulmonary function assessments

Pulmonary function tests were performed at the Erciyes University Medical Faculty Chest Diseases outpatient clinic by trained and experienced respiratory function test technicians of at least 5 years using a Vmax 20c spirometre device while the patients were at a sitting position. Following at least 3 acceptable maneuvers of the pulmonary function test, the best test was recorded. FEV_1_ (Forced expiratory volume in 1 s), FVC (forced vital capacity), FEV_1_/FVC, FEF25-75 values were measured and presented as the percentage of the expected value, according to the patient’s age and height. 

### 2.4. Statistical analysis

Data were entered into Statistical Package for Social Sciences software v: 17.0 (SPSS Inc; Chicago, IL, USA), and analyses were made using the same software program. The distribution of continuous variables was tested with the one-sample Kolmogorov– Smirnov test, and the data are shown as mean ± standard deviation or median and minimum–maximum intervals. For all parametric variables, between group comparisons were made by using repeated measures Anova. For all nonparametric variables, between group comparisons were made by Friedman test, and Dunnett’s multiple comparison test was made within groups when the difference was statistically significant. A p value of < 0.05 was considered to be significant in all analyses.

## 3. Results

This study included 41 severe asthmatic subjects [nine males (22%); mean age 48.8 ± 10.6; mean duration of mOCS treatment 5.1 ± 4.0 months]. Baseline demographics were presented in Table 2. Mean ACT scores were 16.6 ± 4.8 points at baseline. FEV_1_ and FEF25-75 values before mepolizumab treatment found as 78.9 ± 23.2%, and 45.1 ± 23.1%, respectively. Of the 41 patients, 35 (85.4%) were receiving mOCS therapy before mepolizumab, with a median dose of 4 mg. The median eosinophil count at baseline was 450 (min-max, 10-2460) cells/µL.

**Table 2:** Characteristics of the patients.

	N = 41
Female sex n (%)	32 (78%)
Age, years, mean ± SD	48.8 ± 10.6
Smoking n, (%)	
Never smoked	39 (95.1%)
Ex-smoker	1 (2.4%)
Active smoker	1 (2.4%)
Asthma duration, years, mean ± SD	11.2 ± 5.8
Mean follow-up duration, years ± SD	5.1 ± 1.9
Methylprednisolone equivalent systemicsteroid dose before mepolizumab, mg, median (min-max)	4 (0–16)
Nasal polyps, n (%)	22 (53.6%)
NERD, n (%)	16 (39%)
Atopy, n (%)	18 (43.9)

NERD: NSAID-exacerbated respiratory disease.

### 3.1. Clinical efficacy of mepolizumab treatment on severe eosinophilic asthma

Of the patients, 34/41 (83%) were continued the mepolizumab treatment after 12 weeks. Seven patients were not included in the 12 weeks assessment for mepolizumab efficacy [5 were awaiting 12 weeks assessment, one stopped due to adverse drug reaction (serum-sickness like disease), one stopped due to difficulty in obtaining the drug before 12thweek] (Figure 2). When comparing the change in blood eosinophil counts, mOCS doses and ACT scores between baseline and at week 12 under mepolizumab treatment, a marked decrease in peripheral eosinophil counts (3.7 (0.1–18)% vs. 1.3 (0.2–2.8)%; p < 0.001) and an increase in ACT scores (17 (7–25) vs. 23 (14–25); p < 0.001) were observed. At week 12, all of the patients were classified as treatment responders according to increased ACT scores and decreased peripheral eosinophil counts or decreased OCS dose without clinical deterioration. mOCS dose was decreased in 27 of the 34 patients (79.4 %); oral corticosteroids were completely withdrawn in five of the 34 patients. No marked changes in FEV_1_ values were observed at this time point (78.9 ± 23.3% vs 82.5 ± 23.7%).

A total of 31/34 (91%) patients continued the mepolizumab treatment after 24 weeks. Three patients were not included in the 24 weeks’ assessment for mepolizumab efficacy [two were nonresponders (6%), one stopped due to adverse drug reaction in the “possible category” (heart failure) before the 24th week] (Figure 2). Of the responders, 31/33 (94%) were still responders at the 24th weeks’ assessment. After the 24th week under mepolizumab treatment, the decrease in blood eosinophil counts (baseline eosinophil count: 3.7 (0.1–18); 24th week eosinophil count: 1.2 (0.2–4.7)%; p < 0.001) and improvement in ACT scores (baseline ACT: 17 (7–25); 24th week ACT: 24 (15–25); p < 0.001) were continued. mOCS dose was additionally reduced in 15 patients when comparing to 12th week results. Daily oral corticosteroid dose was withdrawn in 4 additional patients at week 24. When compared to baseline, at week 24, a significant decrease in the exacerbation rates within the last 24 weeks was observed (1 (0–8) vs. 0 (0–0); p < 0.001).

A total of 21/31 (68%) patients continued mepolizumab treatment after 52 weeks. Ten patients were not included in the 52 weeks’ assessment for mepolizumab efficacy (9 were awaiting 52 weeks assessment, one was nonresponder before 52nd weeks) (Figure 2). Of the responders, 21/22 (95%) were still responders at the 52 weeks’ assessment. After 52 week under mepolizumab treatment, the decrease in blood eosinophil counts (baseline eosinophil count: 6.2 ± 6.1%; 52nd week eosinophil count: 1.2 ± 0.8%; p < 0.001) and improvement in ACT scores (baseline ACT: 17 (7–25); 52nd week ACT: 24 (17–25); p < 0.001) continued. mOCS dose was additionally reduced in five patients when comparing to 24th week results. In total, the OCS dose of 18/21 (85.7%) patients could be reduced at the end of 52 weeks. mOCS treatment was withdrawn in four additional patients at week 52. In total, 11/21 (52%) patients were able to discontinue mOCS at the end of 52 weeks. At this time point a significant decrease in the exacerbation rates within the last 52 weeks was observed when compared to baseline (1 (0–8) vs. 0 (0–3); p < 0.001). Comparison of mOCS dose, number of asthma exacerbations, ACT scores, and peripheral blood eosinophils at the beginning of mepolizumab and at 12th, 24th, and 52th weeks after treatment was shown table Table 3. 

**Table 3:** Comparison of the clinical, laboratory and functional parameters at the baseline, 12th, 24th, and 52nd week.

	Pre-mepolizumabn = 41	Mepolizumab 12th week n = 34	Mepolizumab 24th week n = 31	Mepolizumab 52nd week n = 21	p
*Methylprednisolone equivalent systemic steroid dose, mg, median (min-max)	6 (0–16)	2 (0–8)	2 (0–4)	0 (0–4)	<0.001
*Number of asthma exacerbations in the last 24 weeks, median (min-max)	1 (0–8)	0 (0–1)	0 (0–0)	0 (0–3)	<0.001
*ACT median (min-max)	17 (7–25)	23 (14–25)	24 (15–25)	24 (17–25)	<0.001
*Eos %, median (min-max)	3.7 (0.1–18)	1.3 (0.2–2.8)	1.2 (0.2–4.7)	1 (0.1–3.6)	<0.001
*Eos count, median (min-max)	450 (10–2020)	100 (10–240)	100 (20–470)	80 (10–280)	<0.001
FEV1 %, mean ± SD	78.9% ± 23.2	82.5 ± 23.7	81.9 ± 23.9	78.9 ± 23.5	0.459
FEV1 L/s, mean ± SD	2117 ± 872	2182.1 ± 878.7	2163.6 ± 856.9	1976.5 ± 800.3	0.329
FEF25-75%, mean ± SD	45.1 ± 23.1%	48.8 ± 23.5%	48.7 ± 23.1%	41.0 ± 20.1%	0.160
FEF25-75 mL, mean ± SD	1620 ± 1060	1699 ± 1060	1675 ± 991	1378 ± 846 mLL	0.085

ACT: asthma control test, eos: eosinophil.

### 3.2. Effect of mepolizumab treatment on pulmonary functions in severe eosinophilic asthma

**Figure 3:**
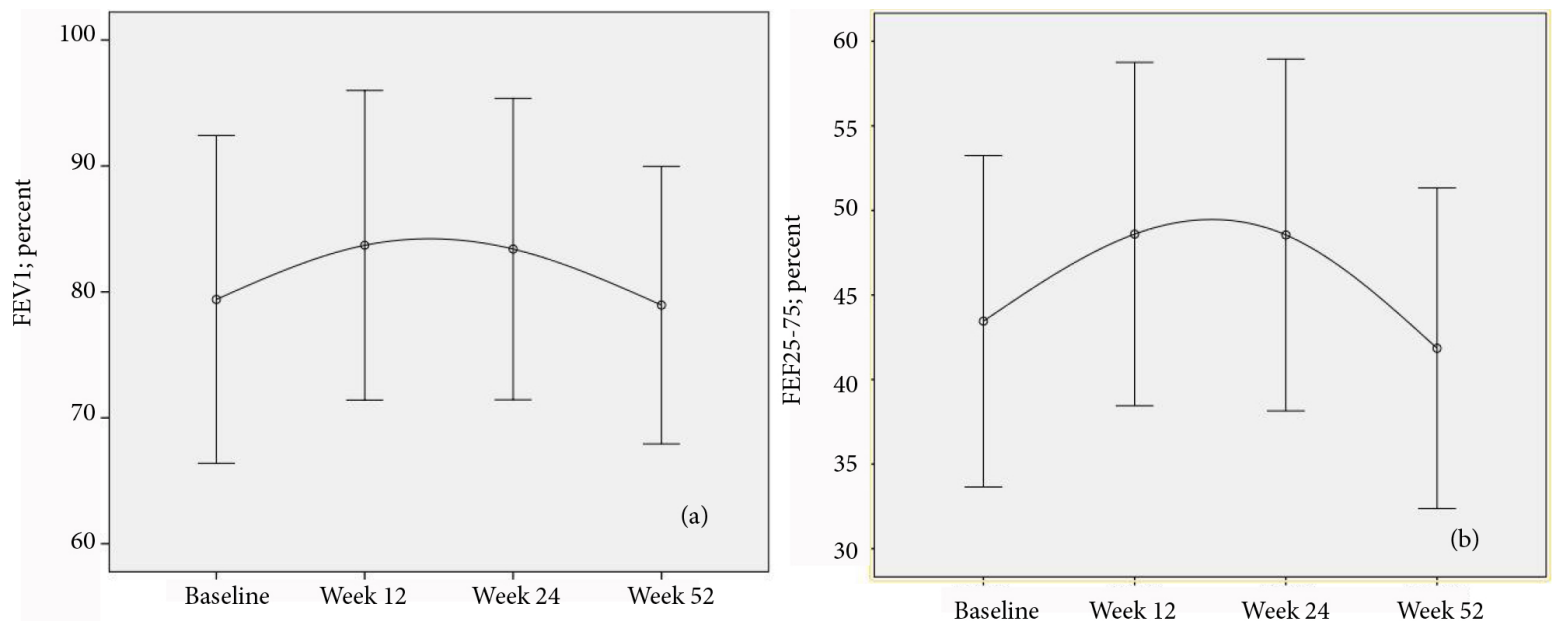
Comparison of FEV1 at the beginning of mepolizumab and at 12th, 24th, and 52nd weeks after treatment.

FEV_1 _values at 12th, 24th, and 52th weeks showed no significant change when compared to baseline values [2228 ± 906 mL (82.9 ± 23.4%), 2163 ± 856 mL (81.9 ± 23.9 %), 1976 ± 800 mL (78.9 ± 23.5 %) vs. 2117 ± 872 mL (78.9 ± 23.2%)]. Also, no marked changes in FEF25-75 values between the baseline and at 12th, 24th, and 52nd weeks were observed [(1699 ± 1060 mL (48.8 ± 23.5%) , 1675 ± 991 mL (48.7 ± 23.1%), 1378 ± 846 mL (41.0 ± 20.1%) vs. 1620 ± 1060 mL (45.1 ± 23.1%)]. Comparison of FEV_1,_ FEF25-75 at the beginning of mepolizumab and at 12th, 24th and, 52nd weeks after treatment was shown in Figure 3a and Figure 3b, respectively. 

## 4. Discussion

Our study showed that mepolizumab therapy reduced the rate of asthma exacerbations, decreased mOCS dose, improved ACT scores, and decreased peripheral blood eosinophil counts in patients with SEA. On the contrary, we found no marked changes in FEV_1 _and FEF25-75 values with 52-week mepolizumab add-on treatment. 

We suggested that SC fixed-dose mepolizumab administration significantly decreased blood eosinophil levels, asthma exacerbations, mOCS doses and improved ACT scores in patients with SEA in agreement with the placebo-controlled studies and real life studies [6–9, 13–15]. There were no significant change in FEV_1_ values after 12, 24, and 52 weeks of mepolizumab treatment, compared to baseline in agreement with some studies [7,15–18]**. **Yet, the important point here is that there was no deterioration in pretreatment FEV_1_ values, and improved ACT scores despite dose reduction or discontinuation of OCS. 

The small airway impairment, as assessed with FEF25-75 and might contribute to long-term persistent asthma and the subsequent risk for poor asthma outcomes, independently of large airway status [19]. Therefore, we evaluated mepolizumab effectiveness on small airways with mid expiratory flow rates. Despite no unanimous consensus on the algorithm to assess small airway function and structure, several noninvasive techniques can detect small airway dysfunction. FEF25-75 is generally thought to be more reflective of small airways obstruction than is FEV_1_ [20,21]**. **Contrary to our expectation, we didn’t find any significant improvement in FEF25%–75% with mepolizumab effect on small airways. However, there was no deterioration in pretreatment FEF25-75 values despite dose reduction or discontinuation of OCS. To the best of our knowledge, there are only two studies evaluating the effect of mepolizumab on small airways in the literature. In the first study conducted by Farah et al, mepolizumab could significantly improve small airways in SEA measured with multiple breath nitrogen washout [22]. The improvement in small airway function was associated with asthma control in the study [22]. Unlike this study, the absence of changes in small airways in our study may result from small airway assessment method differences. The nitrogen washout method may be more sensitive than the FEF25-75 measurement in detecting the change in small airways in patients receiving mepolizumab treatment [23,24]. On the other hand, our study cohort differed from this study cohort. At baseline, our patient population were younger and most of the patients was using mOCS, had nasal polyposis and higher FEV_1_ values. In the second study, Sposato et al. showed that FEF25-75 improved after mepolizumab therapy in patients with SEA on the contrary to our results [25]. Although both studies had similar patient cohorts, different results were obtained. As an explanation of this different results might be due to the low number of patients in our study. Another explanation is that the mOCS doses could be reduced rapidly within 3 months in majority of our cases because there was a significant increase in the symptom scores of the patients and the absence of asthma exacerbation. This decreasing continued until the end of the 52nd week. Therefore, while the dose of OCS can be reduced, we also reduce the improving effect of OCS on small airways. 

The limitation of the present study was its retrospective design. Another limitation is that the lack of validity of the FEF25-75 measurement to reflect small airway functions and inflammation [26,27]. However, The FEF25–75 is the spirometric variable most commonly cited as an indicator of small airway obstruction in literature [28]. The small airways were evaluated retrospectively with FEF25-75 values in our study. If it was a prospective study and our primary aim was to evaluate small airways, we could make a comparison using one of the other methods to evaluate small airways. In this way, we could more clearly evaluate the effect of mepolizumab on small airways.

In conclusion, mepolizumab has been shown to be effective in reducing exacerbations and daily doses of mOCS in this real-life cohort of patients with SEA. Although we found no improving effect of mepolizumab therapy on small airways assessed with mid expiratory flow rates there was no significant deterioration compared to pretreatment FEF25-75 values, and there were improved ACT scores despite dose reduction or discontinuation of mOCS. Further studies comparing the effectiveness of mepolizumab treatment on the small airways with different techniques are needed. 

## Informed consent

All patients were under follow-up at our asthma outpatient clinic provided written informed consent.
